# Implications of remote monitoring Technology in Optimizing Traditional Self-Monitoring of blood glucose in adults with T2DM in primary care

**DOI:** 10.1186/s12902-021-00884-6

**Published:** 2021-11-10

**Authors:** Alex R. Montero, David Toro-Tobon, Kelly Gann, Carine M. Nassar, Gretchen A. Youssef, Michelle F. Magee

**Affiliations:** 1 Department of Medicine, MedStar Georgetown University Hospital, 3800 Reservoir Rd, Washington, DC, 20007 USA; 2 MedStar Diabetes Institute, 100 Irving Street, NW # 4114, Washington, DC, 20010 USA; 3 Mayo Clinic, Division of Endocrinology, 200 1st Street NW, Rochester, MN 55905 USA; 4 BioTelemetry, 1000 Cedar Hollow Road, Suite 102, Malvern, PA 19355 USA; 5 MedStar Health Research Institute, 6525 Belcrest Road, Suite 700, Hyattsville, MD 20782 USA; 6 Georgetown University, School of Medicine, 3900 Reservoir Rd NW, Washington, DC, 20007 USA

**Keywords:** Blood glucose meter, Self-monitoring of blood glucose, Diabetes care management, Remote glucose monitoring

## Abstract

**Background:**

Self-monitoring of blood glucose (SMBG) has been shown to reduce hemoglobin A1C (HbA1C). Accordingly, guidelines recommend SMBG up to 4–10 times daily for adults with type 2 diabetes (T2DM) on insulin. For persons not on insulin, recommendations are equivocal. Newer technology-enabled blood glucose monitoring (BGM) devices can facilitate remote monitoring of glycemic data. New evidence generated by remote BGM may help to guide best practices for frequency and timing of finger-stick blood glucose (FSBG) monitoring in uncontrolled T2DM patients managed in primary care settings. This study aims to evaluate the impact of SMBG utility and frequency on glycemic outcomes using a novel BGM system which auto-transfers near real-time FSBG data to a cloud-based dashboard using cellular networks.

**Methods:**

Secondary analysis of the intervention arm of a comparative non-randomized trial with propensity-matched chart controls. Adults with T2DM and HbA1C > 9% receiving care in five primary care practices in a healthcare system participated in a 3-month diabetes boot camp (DBC) using telemedicine and a novel BGM to support comprehensive diabetes care management. The primary independent variable was frequency of FSBG. Secondary outcomes included frequency of FSBG by insulin status, distribution of FSBG checks by time of day, and hypoglycemia rates.

**Results:**

48,111 FSBGs were transmitted by 359 DBC completers. Participants performed 1.5 FSBG checks/day; with 1.6 checks/day for those on basal/bolus insulin. Higher FSBG frequency was associated with greater improvement in HbA1C independent of insulin treatment status (*p* = 0.0003). FSBG frequency was higher in patients treated with insulin (*p* = 0.003). FSBG checks were most common pre-breakfast and post-dinner. Hypoglycemia was rare (1.2% < 70 mg/dL).

**Conclusions:**

Adults with uncontrolled T2DM achieved significant HbA1C improvement performing just 1.5 FSBGs daily during a technology-enabled diabetes care intervention. Among the 40% taking insulin, this improvement was achieved with a lower FSBG frequency than guidelines recommend. For those not on insulin, despite a lower frequency of FSBG, they achieved a greater reduction in A1C compared to patients on insulin. Low frequency FSBG monitoring pre-breakfast and post-dinner can potentially support optimization of glycemic control regardless of insulin status in the primary care setting.

**Trial registration:**

Trial registration number**:** NCT02925312 (10/19/2016).

**Supplementary Information:**

The online version contains supplementary material available at 10.1186/s12902-021-00884-6.

## Background

In the United States, the increasing prevalence of type 2 diabetes mellitus (T2DM) drives a rising national health and economic burden, now estimated at $327 billion [1]. Self-monitoring of blood glucose (SMBG) is a systematic approach to glucose monitoring which is key to the identification of glycemic patterns to guide timely adjustments in lifestyle and medications -referred to as “pattern management”- to support safe and effective attainment of glycemic targets among adults with T2DM [2]. SMBG’s role in the management of persons not on insulin or agents with hypoglycemic potential has been debated since previous evidence suggests that while there is modest improvement in glycemic control in the short term (6 months), there may be less clear differences in glycemic control in this population in the long term (≥12 months) [3–6]. However, among adults diagnosed with diabetes in the United States, 14.9% take insulin alone, 14.1% take both insulin and oral antihyperglycemic agents and 51.7% take oral medications alone, including insulin secretagogues [7], representing a very large number of patients taking insulin or insulin secretagogues. For this patient population there is evidence supporting the effectiveness of SMBG as a tool to enable successful diabetes care management, individualize glycemic control and prevent diabetes associated morbidity, including hypoglycemia risk and mortality [2, 5, 8–10]. Behavioral research has demonstrated that the effectiveness of SMBG as a health-related tool can be enhanced when it is accompanied by patient education, skills training, structured data feedback and timely titration of antihyperglycemic medications [6], as was the case in many of the pivotal human insulin trials for type 2 diabetes and in the Action to Control Cardiovascular Risk in Diabetes (ACCORD) Study [11–13].

Traditionally, SMBG has been conducted via finger-stick blood glucose (FSBG) monitoring. More recently, continuous glucose monitoring (CGM) has proven to be an alternative and beneficial method of SMBG in type 1 diabetes (T1DM) [14]; yet, its benefits in patients with T2DM are not as well established. A 2019 systematic review with meta-analysis reported that the use of CGM in T2DM was beneficial [15]. Furthermore, although CGM use has increased in adults with T2DM, there are still multiple barriers to its widespread use in this population [16], and its uptake in primary care practices remains low [17, 18]. Therefore, evidence to guide best practices for frequency and timing of FSBG monitoring in adults with uncontrolled T2DM receiving care in primary care settings is still needed.

Telemedicine uses telecommunications to diagnose and treat patients at a distance [19, 20]. Connected health devices (CHDs) generate physiologic data that is transmitted to a smartphone, tablet, or the cloud [21]. Telemonitoring uses CHDs to record patient’s physiologic data for review by health care providers remotely. Technology-enabled interventions, including those which use CHD to perform SMBG, have been proposed as tools to support improved T2DM care management goals [22]. These technologies offer the potential to support a timely integration between SMBG and health care team services, thus enabling patients to safely reach glycemic control targets while potentially reducing acute care services utilization. Telemedicine, mobile app-based, and remote tele monitoring interventions for diabetes have been evaluated in systematic reviews, where they in aggregate modestly lower HbA1C levels [23–25]. Additionally, a previous meta-analysis of remote SMBG interventions in adults with T2DM also found a modest impact on HbA1C (mean difference of − 0.42%; 95% CI: − 0.56% to − 0.27%). Of note, the blood glucose meters (BGM) used in these studies required a patient-dependent data synchronization step through either: a) a hard connection to a modem, computer, or tablet; or b) a Bluetooth connection to a home modem or smartphone [26], and a personal internet connection or telephone line.

Interventions encompassing SMBG and telemedicine have been reported for both primary and specialty care settings [23–25]. We previously conducted a technology enabled T2DM care management “Diabetes Boot Camp” (DBC) intervention which used a novel cellular-enabled finger stick BGM system to support intensified clinical care management and education in the primary care setting. This intervention led to a significant improvement on HbA1C from 11.2 to 8.1% for the intervention group as compared to 11.3 to 9.9% for controls (mean difference of − 3.1 for the intervention group and − 1.6 between groups; *p* < 0.001) [27].

This secondary analysis presents an evaluation of the relationship between the frequency and timing of FSBGs, generated by the BGM during the DBC, and glycemic outcomes. Additionally, it provides insight into the use of a novel CHD that auto-pushed FSBG data to a cloud-based provider dashboard without Bluetooth connection, patient-dependent data synchronization steps, or accrual of data costs to the patient. While BGM in common use becomes increasingly connected and capable of user-friendly remote monitoring, use of traditional FSBG monitoring is still common, particularly in primary care practice. Learnings from this study may be used to inform the evidence base which guides the frequency and timing of FSBG monitoring in primary care settings among adults with T2DM regardless of whether traditional or connected BGM is utilized.

## Methods

### Study population and data collection

In the primary study, the DBC was offered to adults with uncontrolled T2DM (HbA1C > 9%) receiving care in one of five primary care practices in a healthcare system. The intervention included an initial in person diabetes self-management education and support (DSMES) visit followed by 10 weeks of telemedicine visits by physician supervised nurse practitioners and Diabetes Care and Education Specialists (DCES) [27]. Educational material was adapted from the Association of Diabetes Care and Education Specialists (formerly the American Association of Diabetes Educators) ADCES7 Self-Care BehaviorsTM [28]. It covered healthy eating; glycemic targets and SMBG; taking medications as prescribed; hyperglycemia and hypoglycemia recognition, treatment and prevention; knowing when to seek medical help; lifestyle and other topics identified by the participant or the provider. Supplement 1 includes a complete description of the DBC.

During the first in person visit, the participant was registered and trained on SMBG using an FDA cleared BGM (BioTel™ BGM System). This near real-time BGM was capable of automated upload of FSBG values to a cloud-based dashboard via cellular network. The participant simply performed a FSBG check in a procedure that is similar to that used with any standard BGM. There were no additional participant driven synchronization steps beyond performance of the FSBG check. Data transmission occurred immediately if the participant was within cellular range or was delayed then auto pushed once a cellular connection became available. The device manufacturer absorbed the cost of cellular network access and participants did not accrue any financial charges for cellular data use. Participants were asked to check at least two FSBG daily during the DBC, with testing initially requested before breakfast and 2 h after dinner. Timing of the of FSBG checks was varied over the course of the intervention, as needed, to guide medication and lifestyle management decisions.

**Fig. 1:**
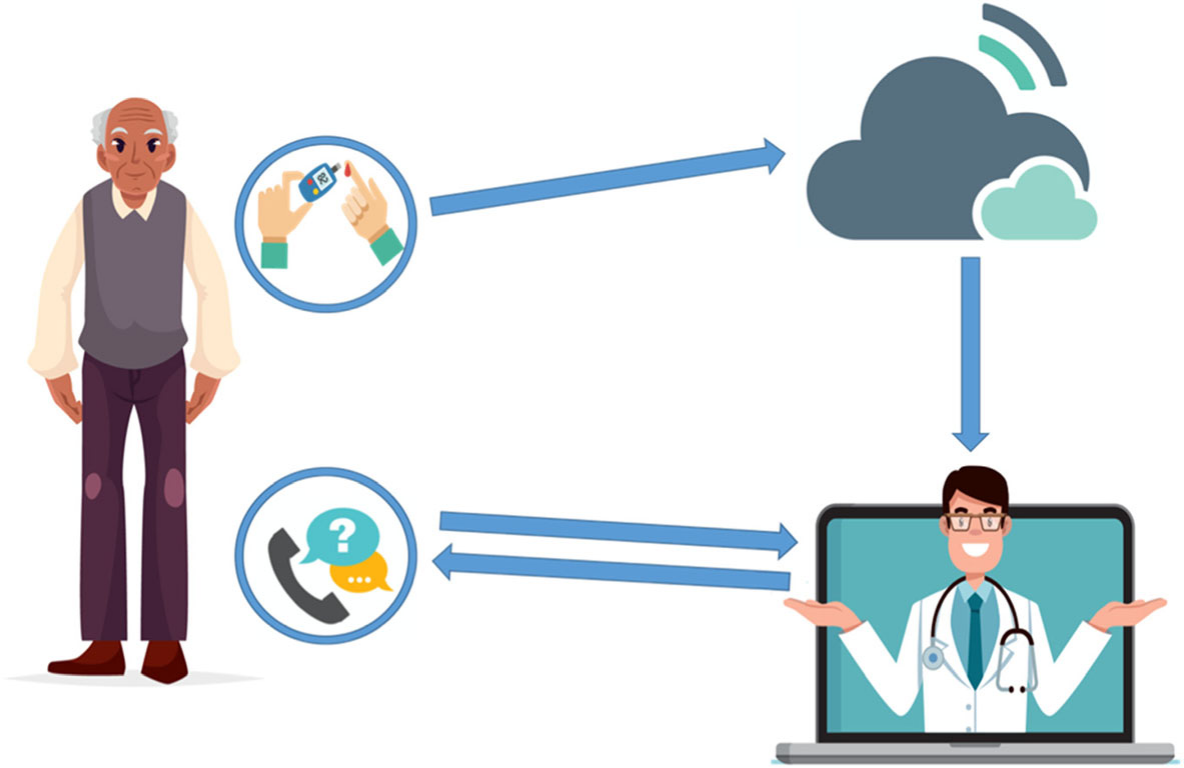
Visual representation of the Diabetes Boot Camp (DBC) workflow. All finger-stick blood values were automatically uploaded to a cloud-based dashboard via cellular networks in near, real-time. The DBC’s team accessed the cloud-based dashboard and conducted a weekly audit of glycemic trends and a daily audit of FSBG extremes. The participant was contacted by the DBC’s team to provide medication titration, guidance, and further education when teachable moments (Including, FSBG extremes) were identified. (Figure was designed and owned by the authors of this manuscript)

Follow up consisted of weekly DBC team review of the individual’s FSBGs on the BioTel™ dashboard. Views included FSBG averages, highest and lowest readings, BG pattern tables by time of day, adherence to the prescribed number of FSBG checks, and extreme highs (> 350 mg/dL) and lows (< 70 mg/dL), the latter of which also generated daily alerts. The DBC team provided tailored DSMES, feedback on BG results, and algorithm-driven medication adjustments (Fig. 1). All participants were informed that active DBC team surveillance occurred only intermittently during office hours and were instructed to contact their primary care provider or go to the emergency department in case of symptomatic glycemic extremes outside office hours.

### Statistical analysis

For this sub-analysis, only the participants from the intervention arm of the parent study were included. The Biotel™ BGM system’s dashboard and the DBC’s team login events were available for analysis. The dashboard’s individual BG checks were used for descriptive analysis which generated the daily mean FSBG checks per participant, the distribution of FSBG checks by time of day, and the prevalence of hypoglycemic episodes (FSBG < 40, < 54 and < 70 mg/dL). Our primary outcome was change in HbA1C from baseline to DBC’s completion 3 months later. Our independent variable was average daily FSBG per participant. Covariates consisted of age, race, insurance status, and insulin treatment status at the end of DBC. Bivariate analyses used T Test or linear regression as appropriate. General linear models were used to assess for an association between average daily FSBG and change in HbA1C, while controlling for all covariates which trended toward significant in bivariate analyses (age, race, and insulin status). Chi square testing was used to assess for a variation in the distribution of FSBG check by time of day throughout DBC follow up (month 1 vs. month 2 vs. month 3).

## Results

A total of 366 participants completed the DBC intervention. Mean age was 56.1 years; 62.6% were female; 79.2% were Black; and 41.8% had Medicaid (Table 1).

**Table 1:** DBC Participants (*n* = 366)

Characteristic		
**Age, mean (SD)**	56.7 (10.6)	
**Female, n (%)**	225 (62)	
**Race**		
White, n (%)	49 (13)	
African American, n (%)	296 (81)	
Hispanic, n (%)	5 (1)	
**Insurance type, n (%)**		
Commercial. n (%)	6 (2)	
Medicaid. n (%)	154 (42)	
Medicare. n (%)	64 (18)	
Private. n (%)	134 (37)	
Self-Pay. n (%)	8 (2)	
**Baseline HbA1C**	11.2 (1.7)	
**Pharmacologic Regimen**		
Regimen Type	**Baseline**	**DBC End (3 Months)**
Any Insulin, n (%)	244 (68.0)	250 (69.4)
Basal-Bolus Insulin	145 (40.4)	145 (40.4)
Mono Basal	95 (25.95)	104 (28.4)
Sulfonylurea	65 (17.8)	40 (10.9)
GLP-1	59 (16.1)	116 (31.7)

A total of 48,111 FSBG values were successfully transmitted to the BioTel™ BGM system cloud-based dashboard. Overall, DBC participants successfully transmitted an average of 1.5 BG values per day (Tables 2 and 3). The DBC team accessed the Biotel™ dashboard an average of 3.9 times per patient per month. Regarding hypoglycemia, of the available FSBG values, 579 were < 70 mg/dL (1.2%), 133 were < 54 mg/dL (0.28%), and 89 were < 40 mg/dL (0.18%). In bivariate analyses, higher average daily FSBG checks, male sex (*P* = 0.02), and no insulin use (*P* = 0.01) were associated with greater improvement in HbA1C. In multivariate analyses (Table 4), higher average daily FSBG checks (*P* = 0.0004) and no insulin use (*P* = 0.001) remained associated with greater improvement in HbA1C. Overall, the most common times of day for FSBG checks were pre-breakfast and post-dinner; and the least common, was overnight (Table 5). The distribution of FSBG checks by time of day changed over time with patients testing more frequently pre-breakfast in the third month of the intervention compared to the first month (*p* < 0.001).

**Table 2:** Finger-Stick Blood Glucose (FSBG) Testing Frequency

Total FSBG checks^a^, mean (SD)	134 (66)
Daily FSBG checks^a^, mean (SD)	1.49 (0.73)

*SD* Standard deviation

^a^Average per participant during the 90 days of the intervention

**Table 3:** Frequency of Finger-Stick Blood Glucose Checks by Insulin Dosing Regimen

Regimen Type^**a**^	n	Daily average FSBG checks^**b**^ Mean (SD)	p
Any Insulin	250	1.56 (0.73)	0.003
Non-Insulin	110	1.32 (0.71)	< 0.005

*FSBG* Finger-stick blood glucose, *SD* Standard deviation

^a^At end of DBC (3 Months)

^b^Average per participant during the 90 days of Boot Camp

**Table 4:** Multivariate analysis

Parameter	Estimate	Standard Error	t Value	Pr > |t|
**Intercept**	−2.907558637	0.71792849	−4.05	<.0001
Average FSBG checks	−0.52375887	0.1453201	−3.6	0.0004
Any insulin vs. No insulin	−0.58564606	0.22544617	−2.6	0.0098
Age	0.015467275	0.01041493	1.49	0.1384
Non-Black vs. Black	0.506415448	0.26152655	1.94	0.0536
Non-Medicare vs. Medicare	−0.190364374	0.28374669	−0.67	0.5027

**Table 5:** Finger-Stick blood glucose checks distribution by time of day

Time Period	n (%)
00:00 to 06:00 (overnight)	3399 (7.1)
06:01 to 09:00 (pre-breakfast)	10,954 (22.8)
09:01 to 12:00 (post-breakfast)	8815 (18.3)
12:01 to 18:00 (post-lunch)	10,945 (22.8)
18:01 to 23:59 (post-dinner)	13,998 (29.1)

## Discussion

Despite the high prevalence of T2DM and its expected worsening burden of disease over the next 20 years [29], primary care providers still face numerous challenges to monitor glycemic trends and adequately guide the management of the more than 90% of patients with T2DM for whom they provide the majority of diabetes care management [17]. The ACP (American College of Physicians) recommends routine SMBG for patients: on insulin; with evidence of hypoglycemic episodes; or, taking drugs likely to increase hypoglycemia while driving or operating machinery. Short-term monitoring is suggested when starting corticosteroids or to confirm suspected hypoglycemia. Neither the timing nor frequency of FSBGs are specified [30].

Anti-hyperglycemic regimen adjustment for T2DM relies on targeting HbA1C levels and data generated from SMBG through traditional FSBG checks and, more recently, by CGM. The use of CGM in T2DM, which has potential to overcome shortfalls in SMBG by finger stick, is slowly increasing, however, it has not yet been widely adopted, therefore FSBG remains the primary modality of SMBG for T2DM patients particularly in primary care settings [16–18]. There are acknowledged limitations to the utility of traditional FSBG data. It examines discrete points in time which may miss critical periods relevant to optimization of glycemic control and does not quantify glycemic variability [18]. Furthermore, FSBG monitoring can be underutilized or unreliably reported by patients [18, 31–33]. As BGM systems in common use become CHDs, remote glucose monitoring will likely become a more common option in primary care settings, particularly as payers increasingly cover this service.

This secondary analysis examined the relationship between FSBG check frequency and HbA1C change in adults with uncontrolled T2DM in the primary care setting. Importantly, from a real-world primary care diabetes care management perspective, in this study the average daily frequency of FSBG checks was low, despite 40% of the participants being on insulin by the end of the intervention. Participants on average performed 1.5 FSBG checks daily. This is a considerably lower frequency than the guideline recommended 4–10 per day for patients on insulin regimens [34]. More importantly, this finding was statistically significant regardless of whether the participant was on insulin or not by the end of the intervention. The robust improvement in HbA1C levels as a result of the intervention suggests that requesting a minimum of two FSBG checks daily (initially pre-breakfast and post-dinner) can generate actionable BG data if the values are reliably transmitted by a CHD and then used to guide glycemic regimen adjustments by the provider in collaboration with the patient, either on a recurring basis or prior to office visits.

From a technology perspective, SMBG via a novel, user-friendly CHD was coupled with telemedicine delivery of targeted DSMES and medication management using telephone calls, texting, and email to enable successful delivery of a diabetes care management intervention to adults with uncontrolled T2DM. Compared with prior studies using remote SMBG [25, 26], this intervention evaluated use of a system capable of auto-transmitting FSBG data to the cloud via cellular networks without any user-dependent synchronization steps and without a need for Bluetooth or a personal internet connection. We hypothesize that from the patient perspective, the lack of required steps beyond performing a FSBG using the device, as one would do with any regular BGM, was responsible to some degree for the success of the intervention. The technology also allowed the care team to access FSBG data on-demand which facilitated a timely response to glycemic extremes, expedient diabetes education and medication adjustments targeted to glycemic patterns and trends.

Although diabetes specific remote tele monitoring interventions are numerous, few rigorous studies have been reported. Moreover, the type of CHDs and telemonitoring technologies deployed are variable and dated, which makes positioning of our findings in the context of the existing literature challenging. Lim et al. reported a Bluetooth-enabled BGM capable of FSBG data transfer via synchronization to a home telephone modem [35]. Similarly to this study, they reported a daily FSBG check frequency of 1.5 per day but a more modest HbA1C reduction (− 0.9). More recently, Quinn et al. reported the results of a Bluetooth-enabled BGM which synchronizes with a smartphone or tablet app and transfers data wirelessly to a cloud-based dashboard if internet connection is available. They achieved a − 1.2 reduction in HbA1C compared to controls; however, data on frequency of BG monitoring was not reported [36, 37].

The degree of improvement in glycemic control reported in prior studies has not been to the same degree as was found in this study. It is possible that the more modest improvements in glycemic control in prior studies were partially attributable to inability to effectively engage patients in the intervention, lack of end-user acceptance of the BGM technology and inadequate access to communications infrastructure [38], as well as significant complexity in BGM synchronization and data transmission processes. In the present intervention, there were no such participant-dependent steps for data transmission. This facilitated a user-friendly experience for both the participants and the DBC team while allowing them to exchange glycemic data seamlessly and reliably in order to guide time sensitive telemedicine care. Additionally, there was no data plan cost to the participants, removing a potential financial barrier to its use, particularly among vulnerable populations such as that served in this study.

While this report focuses on the use of FSBG monitoring via a novel CHD, SMBG can also be performed via CGM. A recent systematic review with meta-analysis on the use of CGM in T2DM compared to FSBG monitoring, demonstrated a statistically significantly greater lowering of HbA1c with CGM [15]. However, the difference in the pooled mean HbA1C levels between T2DM patients on CGM versus FSBG monitoring was modest (mean difference in HbA1C: -0.25; 95% CI: − 0.45 to − 0.06) [15]. While this may be important from a population health perspective, from the pragmatic perspective it would not necessarily be considered clinically significant and did not achieve the magnitude of HbA1C lowering demonstrated in the present study. Moreover, persons with T2DM may face multiple barriers for the adoption of CGM technology such as lack of insurance coverage, high out of pocket cost, patient-dependent data synchronization and transmission processes, device obtrusiveness (e.g. pain, irritation, discomfort or cosmetic disapproval) and alarm fatigue [39]. When future strategies to enable primary care practices to monitor and respond to remote BGM systems can be integrated within usual office workflow, it will be important to factor in features of systems such as those in the one used in this study which might be considered “low-tech” in terms of usability from the patient perspective yet function fully as a “high-tech” CHD.

While the role of the primary care provider in managing patients with T2DM on insulin regimens is growing [17], there is still limited uptake of CGM in this setting [15, 18]. The evidence generated in this study demonstrates that requesting two FSBG values daily and analyzing on average 1.5 daily FSBG results can yield adequate data to guide safe and effective anti-hyperglycemic agent and lifestyle regimen adjustments targeting optimization of blood glucose control in patients with T2DM regardless of their insulin use status. This alternative remote telemonitoring supported SMBG strategy was effective for short term glycemic optimization (12 weeks). Future research will be needed to evaluate its long-term effects.

This study had several limitations. The cohort was predominantly Black, reflecting the demographics of the population cared for in our system, and included adults with uncontrolled T2DM of ages ranging from the 40s to 60s. These features may limit generalizability to other racial, ethnic or younger/older age groups. Although formal cost effective analysis was not conducted, this intervention identified a potential significant reduction in hospitalization related costs [27], and included diverse payers - Medicaid, Medicare and Private insurances- which would support its applicability across a variety of payers. Moreover, the supplies and other out of pocket costs of remote monitoring technology for SMBG do not differ from those of traditional SMBG devices. While we put forward that this technology-enabled approach to T2DM care management was successful for a variety of reasons such as intensified clinical management, glycemic pattern driven education and streamlined SMBG, we did not attempt to identify the impact of individual components of this multifaceted intervention on outcomes. These areas will be the subject of future research efforts.

## Conclusions

In summary, this uncontrolled T2DM clinical care management and education intervention generated evidence that adults asked to check two, and who performed an average of 1.5 FSBG daily, could safely and significantly improve their glycemic control regardless of whether or not they are on insulin. The most common timing of FSBG checks was initially pre-breakfast and post-dinner and was then varied as needed to facilitate pattern management. These findings represent an important primary care SMBG strategy regardless of insulin use among persons with T2DM either not willing or who face barriers to adopting CGM, or who cannot or are unwilling to perform FSBG checks more than twice daily. The elimination of additional steps to transmit BG data beyond those typically needed to perform a FSBG check and assuring no data cost to the user should be considered in efforts to promote uptake and adoption of future technology enabled BGM system interventions.

## Supplementary Information

**Additional file 1.**

## Data Availability

The datasets used and/or analyzed during the current study are available from the corresponding author on reasonable request.

## Abbreviations

SMBG: Self-monitoring of blood glucose
HbA1C: Hemoglobin A1C
T2DM: Type 2 diabetes
BGM: Blood glucose monitoring
FSBG: Finger-stick blood glucose
DBC: Diabetes boot camp
CGM: Continuous glucose monitoring
CHD: Connected health device
