# Supplementary material for: Implications of remote monitoring Technology in Optimizing Traditional Self-Monitoring of blood glucose in adults with T2DM in primary care
Source: BMC Endocr Disord. 2021 Nov 10;21:222. doi: 10.1186/s12902-021-00884-6 (PMC8582211; doi:10.1186/s12902-021-00884-6)
Supplement: Supplementary file 1 — Additional file 1. [file 12902_2021_884_MOESM1_ESM.docx]

**Supplement 1.**

**Diabetes Boot Camp Description and Methods**

The Boot Camp used a team-based approach to promote diabetes self-management education and support (DSMES) and timely, technology-enabled antihyperglycemic medication management by physician supervised nurse practitioners (NP) and Diabetes Care and Education Specialists (DCES). Endocrinologists conceived the programme, designed all aspects of the intervention in collaboration with system Primary Care Providers (PCP), DCESs and patients, developed the diabetes medication management algorithm, provided DCES training and ongoing mentoring for all Boot Camp allied health professional providers. They also provided clinical guidance, as needed, for all aspects of program deployment including glycemic management support for individual participants when requested by a DCES or NP.

Medication management was delivered by the DCES and/or NP following a medication algorithm decision support tool based on national guidelines and an evidence-based algorithm for insulin dose titration. The algorithm guided adjustments based on fingerstick blood glucose (FSBG) levels and current medications. DCESs were trained via a full-day education programme and subsequent coaching with an experienced DCES. Study diabetes-centric physicians and NPs were available as needed to provide advice to the DCES. Embedded hard-stops in the algorithm (eg, persistent marked hyperglycemia and/or hypoglycemia) required consultation with a diabetes specialist. A study clinician reviewed and signed all medication orders daily.

DSMES was initiated during the onsite, one-on-one visits with the DCES then continued by the NPs during the weekly virtual visits. The education was adapted from the American Association of Diabetes Educators. It covered healthy eating; glycemic targets and glucose monitoring; taking medications as prescribed; hyperglycemia and hypoglycemia recognition, treatment and prevention; knowing when to seek medical help; lifestyle and other topics identified by the participant or the provider.

DSMES and medication management was facilitated by an FDA-cleared cellular-enabled blood glucose monitoring system (BioTel™ BGM System), which accrued no data charges and was provided to participants with sufficient test strips to do at least two fingerstick BG checks daily. The FSBG measurements were auto-transmitted to a provider dashboard in near-real-time without a need for additional steps by the participant and were reviewed daily by DCES for hypoglycemic and hyperglycemic events and weekly for participant progress.

**The participant’s Boot Camp experience**

Participants attended two in-person meetings with a DCES within a 2-week period. At the first in-person visit, participants completed the ‘KNOW Diabetes’ knowledge test using a tablet, then were auto-directed to short educational videos that corresponded to their knowledge deficits. DSMES content was also provided in print. Participants were provided with the BGM and taught how to use it. DCES adjusted medications using the algorithm when needed. If a new drug was indicated, a shared decision-making process that outlined the potential risks and benefits of two medication choices was used. At the second in-person visit, DCES discussed recent FSBG levels, continued medication adjustment and provided DSMES. The team also addressed other needs, including psychosocial support, access to medications, and referrals to community resources.

Site visits were followed by 10 weeks of virtual visits by telephone, text and/or email. The DCES contacted participants at minimum weekly to discuss progress, adjust diabetes medications, continue DSMES and coach towards lifestyle goals using motivational interviewing strategies. Participants were also contacted when high or low BG alerts were generated. A final in-person or virtual visit occurred at 90 days at which time patients were referred back to their system provider.

Magee MF, Baker KM, Fernandez SJ, Huang C-C, Mete M, Montero AR, Nassar CM, Sack PA, Smith K, Youssef GA, et al. 2019. Redesigning ambulatory care management for uncontrolled type 2 diabetes: a prospective cohort study of the impact of a Boot Camp model on outcomes. BMJ Open Diabetes Research and Care. 7(1):e000731. doi:[10.1136/bmjdrc-2019-000731](https://doi.org/10.1136/bmjdrc-2019-000731).
